# The complete chloroplast genome sequence of *Styrax zhejiangensis*, a species with an extremely small population

**DOI:** 10.1080/23802359.2018.1507639

**Published:** 2018-10-29

**Authors:** Fengping Dong, Xinhong Liu, Xin Shen, Weixing Sheng, Dongyue Jiang, Yingang Li

**Affiliations:** a Zhejiang Academy of Forestry, Hangzhou, People’s Republic of China;; b Zhejiang Agriculture and Forestry University, Hangzhou, People’s Republic of China;; c Zhejiang Jiande Forestry Bureau, Jiande, People’s Republic of China

**Keywords:** *Styrax zhejiangensis*, chloroplast genome, phylogenetic relationship, conservation

## Abstract

*Styrax zhejiangensis* is an endemic species to China and is only distributed in Jiande, Zhejiang Province. The species is on the verge of extinction. The chloroplast genome of *S. zhejiangensis* was determined from Illumina pair-end sequencing data. The sequence was 157,387 bp and consisted of one large (LSC, 87,193 bp) and one small (SSC, 18,286 bp) single-copy region region, separated by a pair of inverted repeat (IR, 25,954 bp) regions. The sequence included 116 genes, including 82 protein-coding genes, 19 rRNAs and 15 tRNAs. The overall GC content was 37.0%. A maximum likelihood phylogenetic analysis showed that Styracaceae was more closely related to Symplocaceae than to Ebenaceae.

*Styrax zhejiangensis*, one of 234 Styracaceae species, is a broad-leaved tree species with beautiful white flowers (Wu et al., 2013). *S. zhejiangensis* is endemic to Zhejiang Province, China, with an extremely narrow distribution area. At present, only one very small population, with less than 70 adult individuals of *S. zhejiangensis,* has been found at the Jiande Forest Farm. The species is on the verge of extinction. There is not any report of this Endangered species in conservation biology and phylogeny studies, except revisions of some species in the same genus (Gene 1974, Peter 1997, 1999). Chloroplast genomes are widely used in studies of species conservation, genome evolution, and phylogeny (Jansen et al. 2007, Moore et al. 2007). Based on the complete chloroplast genome sequence of *S. zhejiangensis*, analysis of the composition and structure of this chloroplast genome can reveal more information about its genetic variation, eliciting mechanism.

We sampled a single individual of *S. zhejiangensis* for total genomic DNA from the nursery of the Zhejiang Academy of Forestry, China, located at 30.21°N, 120.02°E. High-throughput sequencing was carried out using HiSeq™ 2000 analyzer (Illumina, San Diego, California). All the data were assembled into the genome using SPAdes v3.9.0 (Bankevich et al. 2012). The annotation was performed with the DOGMA (Wyman et al. 2004). Protein-coding genes were identified using the plastid/bacterial genetic code. Intron/exon boundaries were determined using the MAFFT v7 (Katoh and Standley 2013), and tRNA boundaries were verified using tRNAscan-SE with default settings (Schattner et al. 2005). The GC content was measured using Geneious version 8.0.2 (Kearse et al. 2012).

The complete chloroplast genome of *S. zhejiangensis* (GenBank accession number MG702338, https://www.ncbi.nlm.nih.gov/nuccore/MG702338.1/) was 157,387 bp in length, and the circle was typical in its general structure of most plastids with a pair of IRS (25,954 bp) which were separated by a large single-copy region (LSC; 87,193 bp), and a small single-copy region (SSC region; 18,286 bp). The genome encoded 116 functional genes, consisting of 82 protein-coding genes, 19 ribosomal RNA genes, and 15 transfer RNA genes. Among these genes, 15 genes harbored a single intron. 18 genes were duplicated in the IR region. The overall GC content was 37.0%, while the corresponding values of the of IR, LSC, and SSC regions were 42.5, 34.8, and 30.3%, respectively.

To ascertain the phylogenetic placement of *S. zhejiangensis* within the order Ebenales, maximum likelihood analysis was performed using the MEGA6 software (Tamura et al. 2013) with 1000 bootstrap replicates, according to chloroplast genomes of 14 related species with *Pouteria campechiana* as an outgroup. The tree revealed that six Styracaceae species formed a monophyletic clade with 100% bootstrap support,and *Sinojackia* was most closely related to *Melliodendron* and nearer to *Alniphyllum* than to *Styrax* consisting of *S. zhejiangensis* and *S. grandiflora.* Styracaceae was more closely related to Symplocaceae than to Ebenaceae (Figure 1). This newly characterized complete chloroplast genome can provide essential resources for the study of genetic diversity and the conservation of this endangered species.

**Figure 1.**
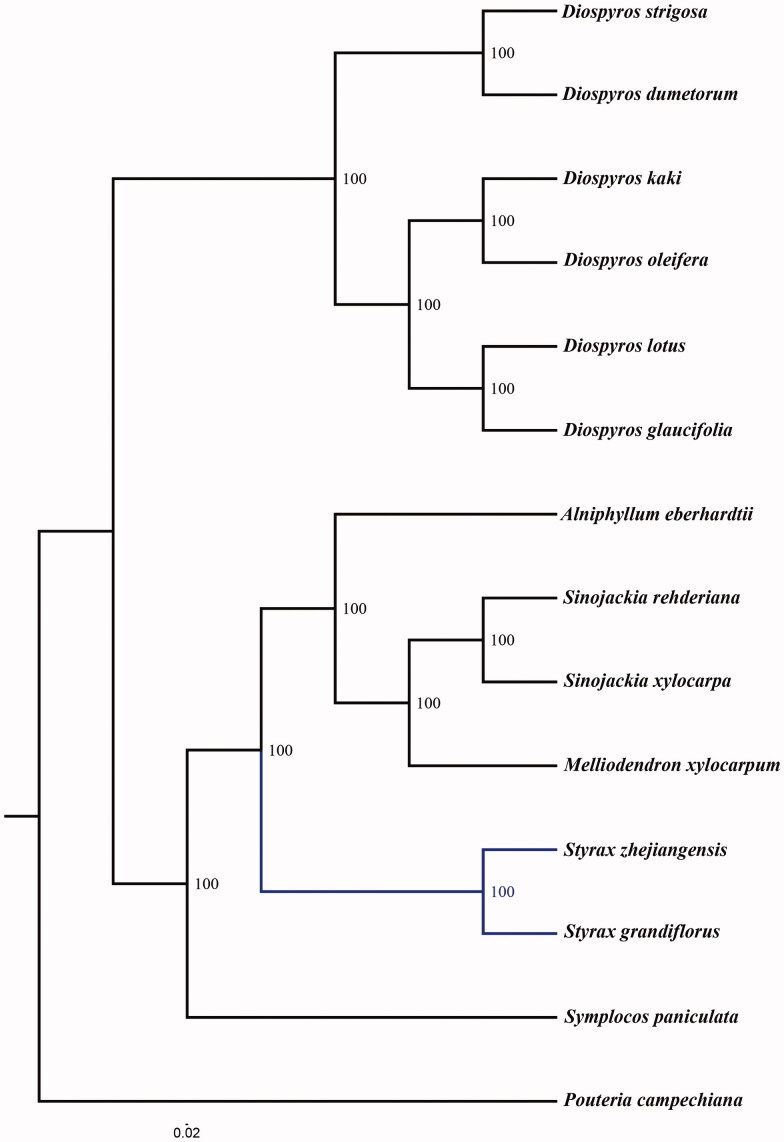
Phylogenetic tree based on 14 complete chloroplast genome sequences. Accession numbers: *Styrax grandiflorus* (KX111381.1), *Alniphyllum eberhardtii* (KX765434.1), *Melliodendron xylocarpum* (MF179500.1), *Sinojackia xylocarpa* (KY709672.1), *Sinojackia rehderiana* (MF179499.1), *Diospyros lotus* (KM522849.1), *Diospyros glaucifolia* (KM504956.1), *Diospyros kaki* (KT223565.1), *Diospyros oleifera* (KM522850.1), *Diospyros dumetorum* (MF179487.1), *Diospyros strigosa* (MF179495.1), *Pouteria campechiana* (KX426215.1), *Symplocos paniculata* (MF179486.1).
